# Extirpation of Lake Sturgeon in an Ontario Lake Following Dam Construction and Watershed Diversion Confirmed by Indigenous Traditional Knowledge and Sedimentary eDNA

**DOI:** 10.1002/ece3.71948

**Published:** 2025-08-08

**Authors:** Stafford Rohtehrá:kwas Maracle, Monica L. Garvie, Calvin Taylor, Michael Fisher, Brian F. Cumming, Stephen C. Lougheed

**Affiliations:** ^1^ Department of Biology Queen's University Kingston Ontario Canada; ^2^ First Nations Technical Institute Tyendinaga Mohawk Territory Deseronto Ontario Canada; ^3^ Ginoogaming First Nation Longlac Ontario Canada; ^4^ Long Lake #58 First Nation Longlac Ontario Canada

**Keywords:** environmental sedimentary DNA, Indigenous knowledge, lake sturgeon, long Lake, Ontario, sediment cores, water diversion

## Abstract

Damming and water diversions for hydroelectricity, flood control, irrigation, and consumption have had profoundly negative consequences for wildlife, ecosystems, and local peoples globally. The assessment and monitoring of ecological impacts have been common practice for only the last half century and are vital in testing population trends as habitats become increasingly fragmented and degraded. Many systems, including the focus of our study, the Upper Kenogami Watershed (UKW), have been subject to large‐scale damming and diversions prior to modern environmental assessments, leaving the consequences largely unknown. Local Anishinaabe communities, Long Lake #58 and Ginoogaming, have long emphasized the many negative consequences for the environment and non‐human kin caused by the Upper Kenogami diversion, including the local extirpation of lake sturgeon from Long Lake. Here we find that the extirpation of lake sturgeon from Long Lake coincided with the construction of the Kenogami control dam, evidenced through Indigenous Traditional Knowledge (ITK) within Ginoogaming and Long Lake #58 First Nations, as well as sedimentary eDNA signatures. We thus show how both ITK and molecular insights together reveal a more compelling understanding of the impacts of freshwater entrainment and damming on the UKW lake sturgeon population. We use this information to suggest what is needed to rebuild sustainable populations and relationships.

## Graphical abstract

Local Anishinaabe communities, Long Lake #58 and Ginoogaming, long emphasized the many negative consequences for the environment and non‐human kin caused by the Upper Kenogami diversion, including the local extirpation of lake sturgeon from Long Lake. We find that the extirpation of lake sturgeon from Long Lake coincided with the construction of the Kenogami control dam, evidenced through Indigenous Traditional Knowledge (ITK) and sedimentary eDNA signatures. We show how both ITK and molecular insights together reveal a more compelling understanding of the impacts of freshwater entrainment and damming on the Upper Kenogami Watershed Lake sturgeon population.

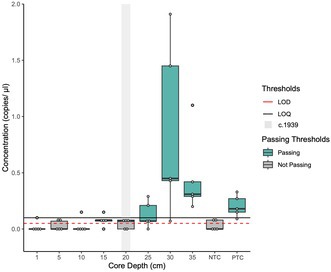

## 1 Introduction

We have seen rapid declines in biodiversity and ecosystems worldwide caused by proliferating human populations and the many resulting ongoing anthropogenic impacts on natural systems (Bar‐On et al. 2018). For example, data from 35,000 monitored populations of 5495 species of fish, amphibians, reptiles, mammals, and birds suggest overall population decreases of 73% globally, with freshwater populations showing the highest declines (85%) versus either terrestrial (69%) or marine (56%) (World Wildlife Fund [WWF] 2024). In Canada, freshwater systems and species are also among the most impacted by human activities. For example, of over 3000 Canadian freshwater species assessed, just under 30% were classified as either at risk (endangered, threatened, or extirpated) or of special concern—with a further ~38% considered data deficient (Desforges et al. 2022). Causes of freshwater biodiversity declines include over‐exploitation, pollution, invasive species, climate change, and habitat loss, fragmentation, and degradation due to drainage, diversions, canalizations, and damming primarily for industrial purposes (Moyle and Leidy 1992; Vaughn 2010; Tickner et al. 2020).

**FIGURE 1:**
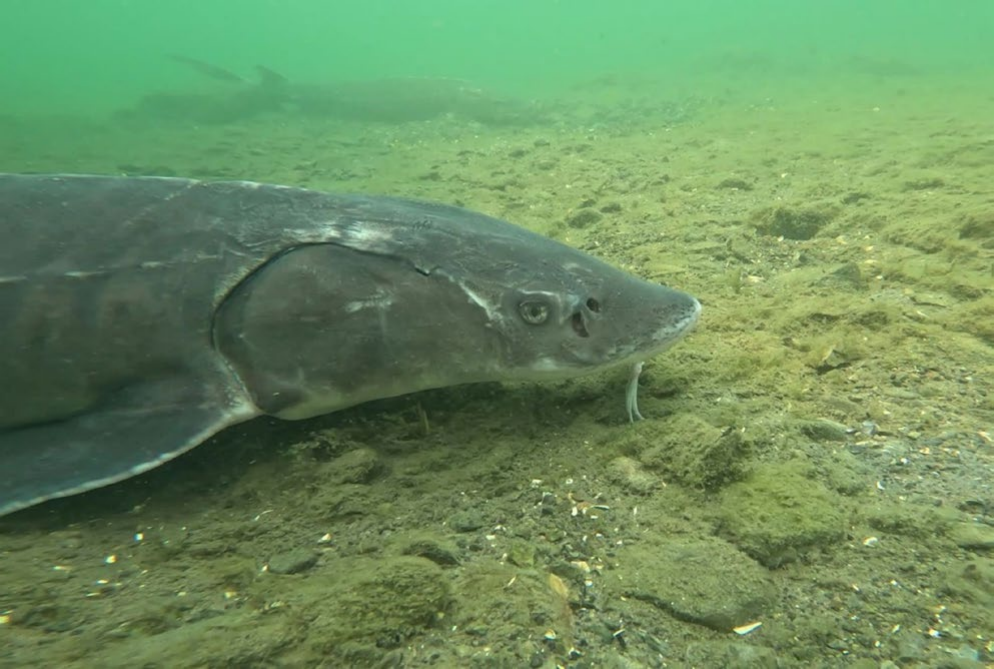
Adult Lake sturgeon (*Acipenser fulvescens*), photo taken by Matthew J. Windle, St. Lawrence River Institute.

**FIGURE 2:**
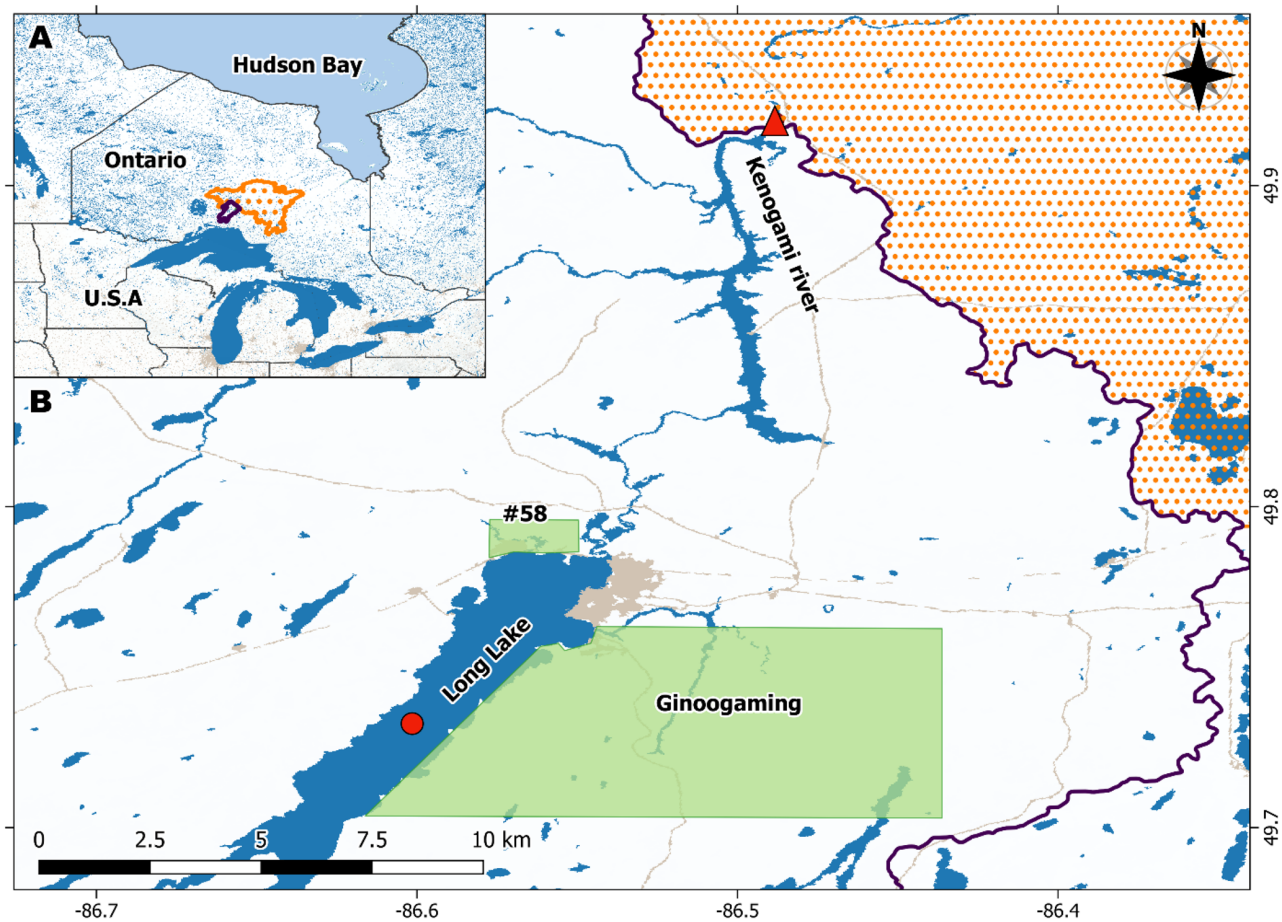
(A) Map of Ontario showing the diverted Long Lake watershed in purple and the Kenogami River Watershed in orange located between James Bay and Lake Superior. (B) Map of the north basin of Long Lake and Upper Kenogami River. The area shaded orange is the current Kenogami River Watershed. The purple line shows the diverted Long Lake Upper Kenogami Watershed (UKW). Green shaded areas are Long Lake #58 and Ginoogaming First Nations territories. The red triangle depicts the location of the Kenogami Control Dam, and the Red Circle depicts the sediment core collection site.

In Canada, over 60% of power generation is hydroelectric, with roughly 240 Terawatt hours (TWh) of a total of 625.7 TWh coming from hydroelectric power generated within the Great Lakes drainage basin (Canada Energy Regulator 2024). Hydroelectricity, along with flood control, irrigation, and general water access for consumption and recreation, often relies on damming for water impoundment or diversions, which can have profoundly negative consequences, not only for many individual species and particularly those that are migratory, but for entire biotic assemblages (Pringle et al. 2000). The extent of these ecological impacts has only been rigorously quantified in the past half century as environmental monitoring and assessments became more common. Such monitoring has been vital in tracking the decline of species and their populations as their habitats become increasingly fragmented and degraded (Kingsford 2000; Morris 2023). However, much of the industrial infrastructure present today was constructed prior to environmental impact assessments (EIA), which began in the 1960s, leaving many ecosystems altered without a scientific understanding of the full scope of the environmental impacts that these habitat alterations have had (Morgan 2012; Wood 2014). Emblematic of this is the Long Lake Diversion and Kenogami Control Dam in Treaty #9 territory, also known as Northern Ontario, Canada (Figure 2). Completed in 1939, the Long Lake Diversion isolated the Upper Kenogami Watershed (UKW), preventing it from naturally flowing north towards Hudson Bay and reversing the flow of the Kenogami River southward through Long Lake/*Ginoo‐gami* ^1^ (as we refer to them in the western or Indigenous context, respectively) into Lake Superior to facilitate the transport of logs to the US market and to increase power generation for the war efforts/manufacturing in southern Ontario and the Canadian market (Peet and Day 1980). Local Anishinaabe communities, particularly Long Lake #58 and Ginoogaming First Nations, were, and continue to be, significantly impacted by the diversion and have long emphasized the many negative consequences for the environment and non‐human kin. Notable among these impacted species was *Acipenser fulvescens* (lake sturgeon or *Namaay* as we refer to them in the western and Indigenous contexts henceforth, respectively; Figure 1), which community members reported disappearing from Long Lake and the other water bodies of the now isolated Upper Kenogami Watershed.

Recent advances in biomonitoring provide new, powerful tools for detecting individual species and quantifying entire assemblages using environmental DNA (eDNA) isolated from multiple environmental media including water, soil, air, and biofilms (Rees et al. 2014; Tournayre et al. 2024). These tools allow us to test for the contemporary presence of species, but also those that existed in the past and may no longer be present via DNA preserved in sediment, permafrost, and ice cores (Willerslev et al. 2004; Thomsen and Willerslev 2015; Sakata et al. 2020). Assays for sedimentary environmental DNA (sedDNA) have been used for over two decades (e.g., Willerslev et al. 2004), but have largely focused on abundant taxa such as bacteria and common benthic invertebrates (Armbrecht et al. 2020; Capo et al. 2021). However, quantitative PCR and digital droplet PCR (ddPCR) methods have enabled the targeting of individual species with low abundance of DNA that comprise a small fraction of the sedDNA, including rare fish species (Nelson‐Chorney et al. 2019; Sakata et al. 2020; Thomson‐Laing et al. 2023; Tournayre et al. 2023). Thus, sedDNA ddPCR methods may allow us to detect species of conservation concern that have been historically undocumented or whose presence remains to be confirmed.

Much like the use of sedDNA, Indigenous Traditional Knowledge (ITK) holds vast empirical and qualitative data on past ecosystems (Kakoty 2018; Jardine 2019). ITK, like Western science, grows and accrues information over time as Indigenous nations worldwide continue to live within and care for the natural environments where they have existed for millennia (Hellier et al. 1999; Stoffle et al. 2003; FiveCrows et al. 2025). Historically overlooked or undervalued in conservation and environmental management, ITK contains within it deep‐rooted empirical insights of environmental changes and shifts in abundance and presence of species spanning centuries, with local peoples' experiences, their cultures, and practices evolving with environmental fluctuations (Hellier et al. 1999; Adade Williams et al. 2020). Many Indigenous nations worldwide have noted the profound negative consequences of human development on local ecosystems, with ITK often unveiling the true extent to which habitat destruction has had on populations of native species and inter‐species relations (Holtgren et al. 2016; Jardine 2019; Morris 2023). Working within frameworks such as the Mi'kmaq Etuaptmumk (Two‐eyed Seeing), co‐produced research that includes ITK and Western science provides a more comprehensive understanding of impacts and informs conservation methods (Kakoty 2018; Maracle et al. 2025). To date, co‐production of knowledge using sedDNA and ITK has been limited to using the latter to simply validate molecular evidence (Lopez et al. 2023). However, ITK can provide far richer insights on and interpretations of the relationships among species and shifting ecosystem functioning, as well as the broader implications for people and the environment that reflect inherently subjective and spiritual interpretations (Kimmerer 2018). This relational wisdom can not only help us to understand how past environments once functioned and how they have changed, but can also guide how we might remediate impacted ecosystems and engage in more sustainable practices to preserve natural ecosystem functions and *anakanahgewein* (rules of traditional governance) (Whyte 2018; Maracle et al. 2025). In the case of the UKW and the Long Lake Diversion, we can draw from sedDNA and local Anishinaabe knowledge (ways and natural laws) to co‐produce and interpret knowledge to understand changing species occurrences for which we lack data, here lake sturgeon, and guide actions to rebuild sustainable populations and relationships.

Lake sturgeon are long‐lived, omnivorous, bottom‐feeding fish found throughout eastern North America (Figure 1). They can take 20–30 years to reach reproductive maturity and only mate every 2–9 years over their roughly 100‐year lifespan, making them extremely sensitive to population decline and diminished recruitment (Billard and Lecointre 2000; Reynolds 2003; Kjartanson et al. 2023). The current range of lake sturgeon encompasses the Mississippi, Great Lakes, and Hudson Bay‐James Bay watersheds. The Hudson Bay‐James Bay watershed was recolonized around 9500 years ago and formed a secondary contact zone between the Mississippian (Great Lakes and southern population) and Missourian (Western Canada) ancestral lineages (Ferguson and Duckworth 1997; Kjartanson et al. 2023). Lake sturgeon typically spawns in gravel beds of shallow fast fast‐flowing waters often below rapids or waterfalls before migrating up to 400 km back to deeper lakes and rivers for foraging (Seyler et al. 2011). Following European colonization, the overall lake sturgeon population declined precipitously throughout much of their historical ranges (including the larger Kenogami Watershed) due to overexploitation (including for caviar), habitat alteration (including damming for hydroelectric power), and pollution (Pollock et al. 2015). Lake sturgeon first received conservation recognition in Canada in 2006; the Southern Hudson Bay‐James Bay population, which includes the UKW, was reassessed in 2017 and remained listed ‘as special concern’ by the Committee on the Status of Endangered Wildlife in Canada (COSEWIC 2017; Seyler et al. 2011). The dire state of lake sturgeon populations has long been known by Indigenous communities, although these insights have not been reflected in the wider scientific literature or conservation prioritization.

Here we show how both ITK and molecular ecological tools together reveal a more comprehensive and compelling understanding of the impacts of freshwater entrainment and damming on the UKW lake sturgeon population. The impacts of the Long Lake Diversion project on the *Namaay* population of the UKW are well known to the community members of Ginoogaming and Long Lake #58 First Nations (C. Taylor Sr., pers. comm. Feb 4 2025; M. Fisher, pers. comm. March 16 2025). The traditional migration route of *Namaay* along the Kenogami River to spawning beds in Long Lake (as well as several other water bodies in the UKW) historically supported a thriving local breeding population as part of the Hudson Bay regional population prior to the diversion project in 1938 (C. Taylor Sr., pers. comm. Feb 4 2025; M. Fisher, pers. comm. March 16 2025; Figure 2A). The limited scientific documentation of lake sturgeon in Long Lake is in marked contrast with the local Anishinaabe knowledge (Peet and Day 1980; C. Taylor Sr., pers. comm. Feb 4 2025; M. Fisher, pers. comm. March 16 2025). Knowledge of the historically abundant population has been and continues to be maintained through oral traditions in the local First Nations communities while community members on the land (e.g., fishers, trappers, harvesters) continue to monitor depleted *Namaay* populations spawning in nearby water bodies (such as Chipman Lake) still connected to Hudson Bay, and specifically the small population spawning just below the Kenogami Control Dam where individuals are physically halted from continuing their migration into Long Lake and the UKW (C. Taylor Sr., pers. comm. Feb 4 2025; M. Fisher, pers. comm. March 16 2025). Honorable harvesting (the sustainable harvesting of non‐human kin following cultural methods and philosophy; see Kimmerer 2018) of *Namaay* by the local community is currently highly restricted due to limited population and spawning locations (C. Taylor Sr., pers. comm. Feb 4 2025; M. Fisher, pers. comm. March 16 2025). Potential impacts on local biota were suggested by Peet and Day (1980) 40 years after the diversion project. Beyond this study (and a series of Ontario Power Generation land studies from the 1990s and 2010s focusing on shoreline erosion in the communities of Ginoogaming and Long Lake #58 First Nations) there have been no funds or efforts to further investigate or remediate the impacts of the diversion on the ecosystem or lake sturgeon population (C. Taylor Sr., pers. comm. Feb 4 2025). We assess the occurrence of lake sturgeon in Long Lake over the last 120 years, encompassing both pre‐ and post‐diversion periods using sedimentary eDNA with a targeted ddPCR assay prompted by consultation with the local First Nations community members.

## 2 Methods

### 2.1 Study Site

Long Lake is a long (74 km), narrow (3 km at the widest point), and deep (200 m maximum depth) lake situated in the Upper Kenogami Watershed (UKW) of Treaty #9 territory in northern Ontario (Figure 2a). Long Lake rests on Canadian Shield Precambrian granite to granodiorite bedrock at an elevation of 311–313 m above sea level (Kresz and Zayachivsky 1991; Ontario Power Generation 2025). The UKW encompasses 4,358 km^2^ of mixed forest and swamp, 24% of which has been logged or otherwise disturbed by resource extraction (Ontario Watershed Information Tool, accessed March 18 2025). The Kenogami River Control Dam is located in the Kenogami River, 16 km north of Long Lake (Figure 2b). Sediment cores (see below) were collected on June 13, 2022, from the north basin of Long Lake at 49°43′45.9″N, 86°36′22.4″W, west of Ginoogaming First Nation and south of Long Lake #58 First Nation (Figure 2b).

*Ginoo‐gami* is an ancestral lake and has been a significant part of Indigenous life and spiritual health since time immemorial. As a meeting place, travel route, and homelands for several *dodem* (clan) families, *Ginoo‐gami* has many legends, stories, and provides teachings based on key components of cultural life/natural laws for Ginoogaming, Long Lake #58, and other First Nations in the region. The Long Lake basin contains the headwaters for the UKW (and is valued as a source of life) and contains pipestone (shale) and other materials for creating subsistence and ceremonial items. Several sites on and around *Ginoo‐gami* are sacred including ceremonial fasting sites, places of offerings for safe passage, and more. Additionally, the shores of *Ginoo‐gami* are the resting place of countless ancestors from countless generations (despite only a few burial grounds noted in western historical documents) and as such require great care.

### 2.2 Indigenous Traditional Knowledge (ITK)

Through ongoing discussions with *Ginoo‐gami* community members for her larger Ph.D. research, co‐author M. Garvie noted community concerns about the disappearance of *Namaay* from *Ginoo‐gami* following the Long Lake Diversion project. These initial concerns were followed up with further discussions and knowledge sharing, ultimately leading to the collection of sediment for environmental DNA analysis. Following our ddPCR analyses, there were further meetings with the Matawa Four Rivers Group, Ginoogaming, and Long Lake #58 environmental departments and land guardians to discuss the interpretation of the results and the implications for *anakanahgewein* (rules of traditional governance) and the restoration of relationships. To include the communities' ITK and support community voice, a knowledge holder from each community with close ties to the land co‐authored this work to discuss the historical and current knowledge of *Namaay* in *Ginoo‐gami* and the UKW, as well as discussing ways to action the knowledge to restore *Namaay* in presence and relationship. ITK shared by the co‐authors and knowledge holders, Calvin Taylor Sr. and Michael (Mikey) Fisher, is cited accordingly following (Macleod 2021), supplemented by knowledge from co‐lead author Monica Garvie.

Calvin Taylor Sr. is Ojibwe and a member and Land & Resources Coordination Officer of Ginoogaming First Nation who holds a deep connection to and immense knowledge of the *Ginoo‐gami* region through his ancestral *tazhiikaaywin* (active presence, ceremony, and subsistence gathering practices of a *dodem* area). Mikey Fisher (*Binesiwin*) is Anishinabe^2^ and a member and council member of Long Lake #58 First Nation, where he maintains an active relationship with the land through traditional trapping and fishing practices as well as ongoing involvement with several *Namaay* preservation and restoration efforts in the *Ginoo‐gami* region. Monica Garvie (*Waasabiikwe*) is Anishinaabe from Hearst, Ontario, 210 km east of *Ginoo‐gami*, and relocated to Longlac on the shores of *Ginoo‐gami* in 1990. Raised with stories, land‐ and community‐based knowledge, she further maintains knowledge of *Ginoo‐gami* and the impacts of the Long Lake Diversion.

### 2.3 Sample Collection

Following Anishinaabe protocols and well‐established paleolimnological methods, two ~35 cm long sediment cores (A1 and A2) were collected from the deepest point of the Long Lake north basin where sediment is expected to pool from the surrounding catchment (high sediment focusing), remain well preserved, and undisturbed by wave action or bioturbation (Wright Jr 1991; Last and Smol 2001; Smol 2009). Sediment was collected using a Glew Gravity core with a ~1 m long, 7.62 cm diameter polyvinyl chloride coring tube from a pontoon raft fixed with a winch system to lower a coring tube into the sediments, allowing it to sink into the sediment under its own weight (Glew 1995). A trigger weight was released from the raft sealing the top of the core tube, allowing the enclosed sediment to be pulled from the lake. Sediment was sectioned at 0.5 cm (core A1) and 1.0 cm (core A2) intervals on site for dating and other analyses. At 5 cm intervals from core A2, 7–10 mL of wet sediment was collected from the center of the exposed section (approximately to 1 cm depth) using a sterilized plastic scoop and placed into a 15 mL Falcon tube and immediately placed on ice for eDNA analysis. The edges of the sections were avoided to minimize contamination from the core tube and any vertical smearing. The remaining sediment was either returned to the UKW system immediately or collected in a sterile Fisher sampling bag and stored under the stewardship of M. Garvie at Queen's University PEARL facility for the duration of her Ph.D. research.

### 2.4 eDNA Isolation From Sediment

Environmental DNA was extracted from the sediment samples using a DNeasy PowerMax Soil Kit designed for extracting low‐abundance DNA from soil samples with high inhibitor loads including humic substances following the manufacturer's protocol (Thomson‐Laing et al. 2022; Qiagen, Toronto, Canada). All sediment collected from each section was used in the DNA extraction. DNA extractions were carried out in a dedicated eDNA laminar flow hood that was decontaminated before extraction using UV sterilization for 20 minutes. All surfaces and pipettes were also wiped with a 10% bleach solution. To test whether contamination was introduced during DNA extractions, we included a no‐template control (NTC) consisting of 10 mL ddH_2_O. Following extraction, samples were eluted in 6 mL of ddH_2_O and separated into 3 separate 2 mL aliquots; one aliquot was used for downstream analysis, and the other two have been placed in a –20°C freezer for long‐term storage.

### 2.5 Core Chronology

Sediments from core A1 were analyzed to create a chronology which was then applied to both cores (A1 and A2) based on their close proximity in a relatively flat and deep basin as assessed by a sub‐bottom profiler at the time of sampling. Gamma spectroscopy was used to determine activities of ^210^Pb, ^137^Cs, and ^214^Pb using equipment similar to that described in Schelske et al. (1994). Elemental analysis was determined using inductively coupled plasma mass spectrometry (ICPMS) for concentrations of Al and Ti as indicators of erosion, to help identify changes in water level and enhanced erosion related to the 1939 diversion project (Bertrand et al. 2024).

### 2.6 Lake Sturgeon ddPCR Assay

A primer pair and probe set were adapted for digital droplet PCR (ddPCR) from a qPCR assay developed by Hernandez et al. (2020) for a 179 bp region of the lake sturgeon mitochondrial cytochrome oxidase 1 (COI) gene; LAST_COI_F 5′‐GCTGGCGGGAAACCTG‐3′, LAST_COI_Probe 5′‐FAM‐TACCATTAT‐ZEN‐TAACATGAAACCC‐MGBNFQ‐3′, LAST_COI_R 5′‐TACCATTATTAACATGAAACCC‐3′. Primer/probe specificity was determined in silico using NCBI Primer‐Blast to search the entire nucleotide database, including all fish species (taxaid: 7898), and targeting only *Acipenseriformes* (7899). A further specificity test was conducted in vitro using qPCR and tissue‐derived DNA from co‐occurring species provided by colleagues at the St. Lawrence River Institute, Cornwall. Lake sturgeon DNA was tested against DNA derived from the tissues of 5 species ( *Anguilla rostrata* [American eel], *Amia calva* [bowfin], *Esox masquinongy* [muskellunge], *Esox lucius* northern pike, and *Alosa pseudoharengus* [alewife]). The assay was specific to the target both in silico and in vitro and was optimized for use on the Bio‐Rad QX200 AutoDG Droplet Digital PCR System (BioRad, Saint‐Laurent, QC, Canada, 2025).

eDNA ddPCR assays were done with 5 replicates each with 6 μL of sample eDNA template. A no‐template control (NTC) of autoclaved distilled water and a positive template control (PTC) with 0.4 pg/μL of tissue DNA were included in 5 replicates each. PCR reactions were carried out in 22 μL volumes: 1× reaction concentration of ddPCR SuperMix for Probes (No dUTP) (BioRad, Saint‐Laurent, QC, Canada), 900 nM of each forward and reverse primers, 500 nM of fluorescence probe, 500 nM of bovine serum albumin (BSA), 2 μL of autoclaved distilled H_2_O, and 6 μL of template. Samples were emulsified into 10–20 thousand droplets using the BioRad Droplet generator with manufacturer recommended volumes: 70 μL droplet oil and 20 μL PCR reaction mix, recovering 42 μL of emulsified sample for PCR. Thermocycler conditions were as follows: 95°C (5 min), 45 cycles of 95°C (30 s) and 57°C (60 s), and 98°C (10 min). Samples were incubated at 4°C overnight to improve droplet stability before being analyzed on the droplet reader.

### 2.7 ddPCR Quantification

**FIGURE 3:**
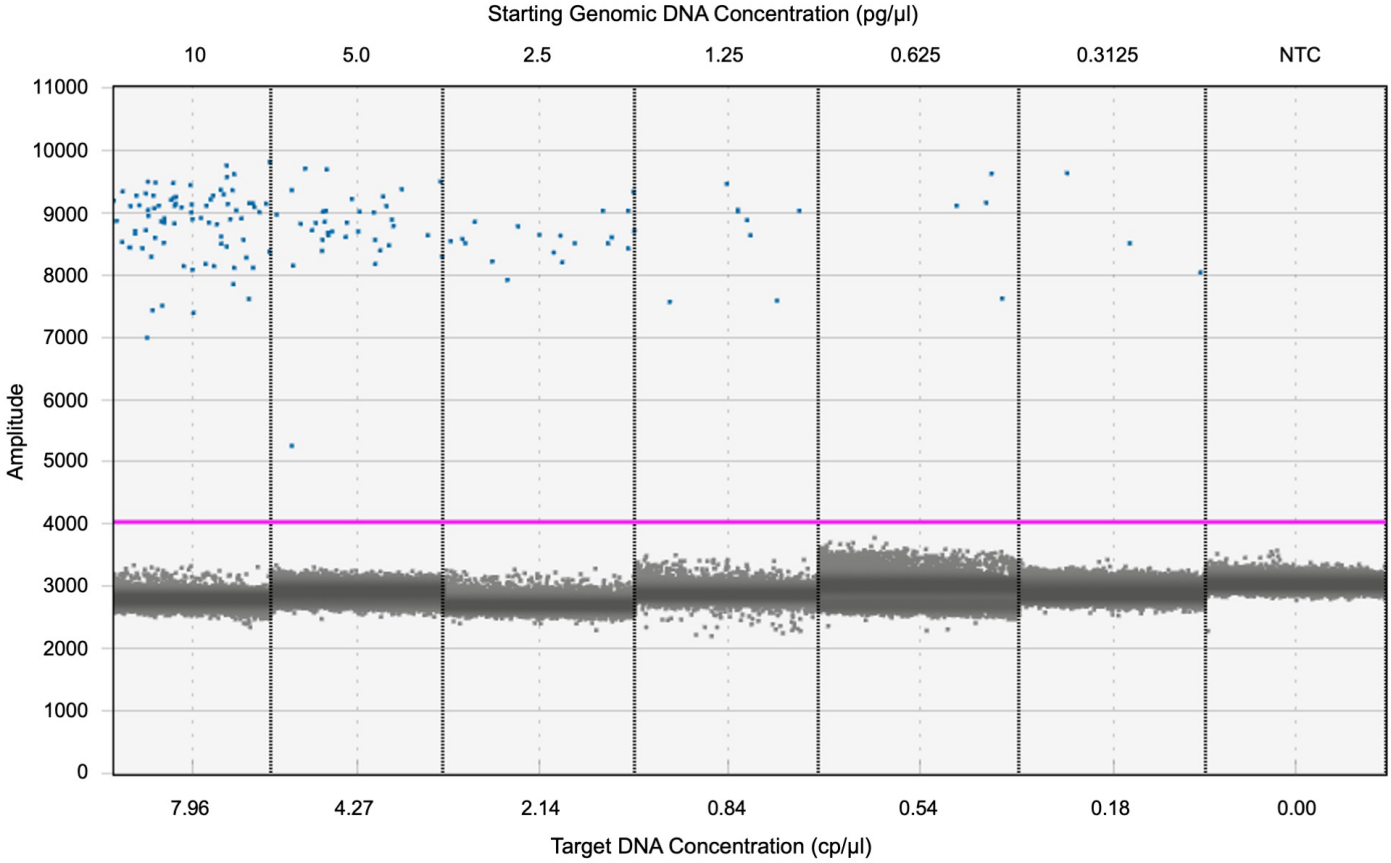
Fluorescence amplitude plot of a 2× dilution series from 10 pg/μL to 0.3125 pg/μL genomic tissue DNA for lake sturgeon. Each concentration was done in triplicate, and one median replicate is shown here. *Y*‐axis shows the fluorescence amplitude of FAM captured in each droplet. Top *x*‐axis shows the starting concentration of each dilution from a 10 pg/μL genomic tissue DNA sample. Bottom *x*‐axis shows the mean concentration (triplicate) of target amplicon in copies per μL (cp/μL) calculated on the ddPCR Quantsoft program from the ratio of positive to negative droplets and well fit with a Poisson distribution. Red line depicts the minimum amplitude threshold for accepting positive droplets. Note—one replicate of the negative control had one determined positive droplet just above the threshold, which was discarded as a false positive.

Following PCR amplification, each droplet was visualized on the QX200 droplet reader for fluorescence of target DNA. Droplets containing target DNA have an increased fluorescence amplitude above the background of negative droplets, which were analyzed using the direct quantification (DQ) mode in the QX Manager 2.0 Standard Edition software (BioRad, Saint‐Laurent, QC, Canada). Fluorescence threshold (minimum positive amplitude) was manually set for each well using the NTC and PTC as references for positive and negative droplet amplitudes (BioRad Droplet Digital PCR Application Guide). Any assays with less than 10,000 droplets were considered inaccurate and discarded. Limit of detection (LOD) and limit of quantification (LOQ) were determined for the assay using a 2× serial dilution from 10 pg/μL down to 0.3125 pg/μL of genomic tissue DNA (10.0, 5.0, 2.5, 1.25, 0.625, 0.3125) (Figure 3). LOQ was determined by finding the lowest standard concentration within the linear range of the standard curve before the concentration plateaus. LOQ was set as 0.1 cp/μL, similar to Brys et al. (2021) and Chen et al. (2023). LOD was qualitatively set as a threshold of at least one positive droplet in three of five replicates or approximately 0.035 cp/μL to control for false positives (Chen et al. 2023; Tournayre et al. 2023). Samples with less than three positive droplets in five replicates were considered a detection but unquantifiable, and samples below the LOD (one droplet in two replicates) were considered to be potentially false positives and a non‐detection.

## 3 Results

### 3.1 Indigenous Traditional Knowledge

Anishinaabe communities in Treaty #9 territory, such as present‐day Ginoogaming and Long Lake #58 First Nations, have had a strong relationship with *Namaay* since time immemorial (C. Taylor Sr., pers. comm. Feb 4 2025). *As* a *nadzookanak* (ancestors) fish, *Namaay* is an integral part of the Anishinaabe clan system whose importance cannot be overstated. The healthy presence of *Namaay* in community life and environment is part of natural law, and it is considered a keystone species which indicates harmony within nature and between humans and the environment (C. Taylor Sr., pers. comm. Feb 4 2025). Anishinaabe cultural stories tell of *Namaay* giving birth and carrying the seven grandfather teachings: wisdom, love, respect, bravery, honesty, humility, and truth (Holtgren et al. 2015). These teachings provide essential spiritual and environmental guidance to the community not only through these teachings and their role in the clan system, but also through observations and interactions such as seasonal indicators on when honorable harvesting is contingent (C. Taylor Sr., pers. comm. Feb 4 2025; M. Fisher, pers. comm. March 16 2025). In addition to spiritual and environmental guidance to the community and the essential contributions to *mino‐biimaadiziwin* (a good life), *Namaay* also provides an essential nutrient‐rich food staple for Indigenous communities (especially elders) in the spring after the long, cold winter months of the boreal region (Holtgren et al. 2015; C. Taylor Sr., pers. comm. Feb 4 2025). Observed stressors which impact (ed) *Namaay* (and many other species) include: construction of the Long Lake Diversion Kenogami Control Dam that acts as a physical barrier to *Namaay* migration into the UKW and *Ginoo‐gami*, the log drive (transportation of logs over water rather than by train or logging trucks) which littered the shores and shallows with abundant debris such as bark fragments and trees (both logs and felled trees) and destroyed *Namaay* habitat and spawning grounds, as well as the increased water levels, “flushes” (artificially prompted pulses of water to flush out logs to the Long Lake route), and erosion which increased turbidity, making habitat unsuitable for *Namaay* and harder to navigate for community members (C. Taylor Sr., pers. comm. Feb 4 2025; M. Fisher, pers. comm. March 16 2025). The Kenogami Dam also impacted *Namaay* on the north side of the dam as the reduced flow destroyed many of the shallow gravel spawning beds (M. Fisher, pers. comm. March 16 2025).

Current community relationships with *Namaay* are impaired by deteriorated population health. For generations, Anishinaabeg in the UKW have honorably and sustainably harvested lake sturgeon and thus maintained an important relationship with this *nadzookanak*. However, as harvesters on the land today, well‐established honorable harvesting protocols are used, and very few *Namaay* are available for community use given the disappearance of *Namaay* in the UKW and drastic population decline north of the diversion (C. Taylor Sr., pers. comm. Feb 4 2025; M. Fisher, pers. comm. March 16 2025). Currently, when caught, *Namaay* must be released to sustain the population; there are simply too few to allow community consumption harvesting (M. Fisher, pers. comm. March 16 2025). This limits the communities' abilities to maintain relationships with this important cornerstone species (C. Taylor Sr., pers. comm. Feb 4 2025).

### 3.2 Assay Specificity

The lake sturgeon in silico test using NCBI Primer‐P‐BLAST search of the entire nucleotide (nt) database revealed no target templates matching the primers and probe. A subsequent search targeting all fishes (taxid: 7898) showed that both primers aligned only to lake sturgeon vouchers with no mismatches, while the probe aligned to multiple *Acipenseriformes* with one base pair mismatch. Further refining the database to only *Acipenseriformes* (taxaid: 7899) showed that both primers and probe align with multiple species with as little as one, two, and one mismatches in the forward, reverse, and probe sequences respectively. This included shovelnose and shortnose sturgeons found in Eastern North America; neither of which are found in the study area nor in the surrounding watershed. The assay did not align with any co‐occurring species and is considered specific to lake sturgeon in this study area—but should not be considered specific where its range overlaps with other sturgeon species. The qPCR specificity assay amplified lake sturgeon tissue DNA successfully and did not amplify DNA from any of the tested co‐occurring species tissue vouchers.

### 3.3 Core Chronology

**FIGURE 4:**
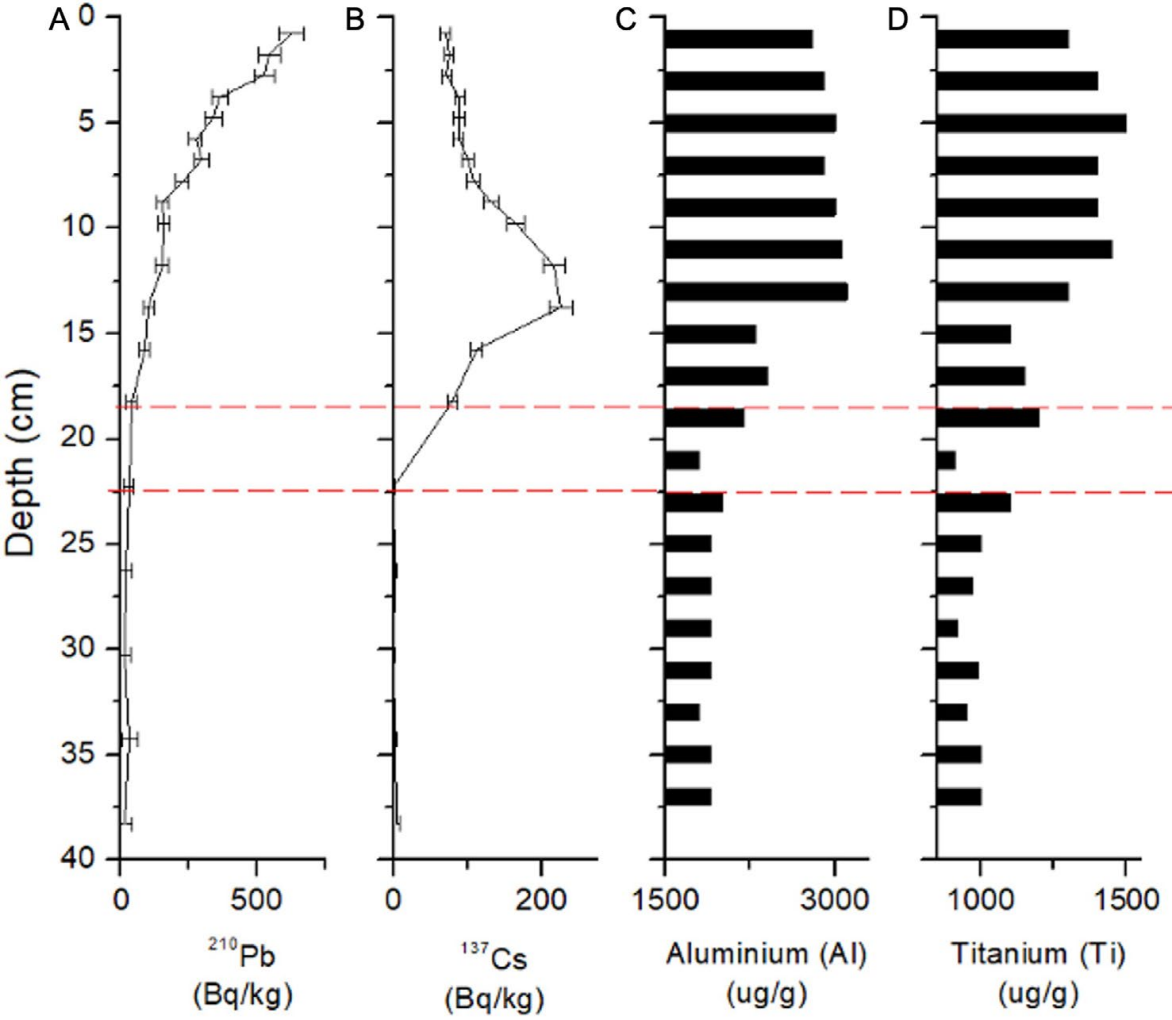
Radioisotope analysis for the 40 cm sediment profile of core A1 using gamma spectroscopy measured in becquerel per kilogram (Bq/kg) for (A) ^210^Pb and (B) ^137^Cs. Elemental analysis of core A1 using inductively coupled plasma mass spectrometry (ICPMS) for the concentration of (C) Al and (D) Ti in micrograms of element per gram of sediment (μg/g) as indicators of erosion. (A) ^210^Pb shows an expected exponential decay curve, confirming minimal mixing of the sediment core. (B) ^137^Cs shows a peak between 11.5 and 13.5 cm in core depth indicating the peak of ^137^Cs fallout associated with atmospheric‐bomb testing in 1963. (C, D) elemental concentrations of aluminum and titanium begin to rise between 19 and 21 cm, consistent with the water‐level rise and increased erosion from the diversion in 1939. Red dashed lines show the approximate depth of the diversion in 1939 based on data from plots (A–D).

Gamma and elemental analyses informed the chronology of core A1, which we assumed was comparable to core A2, as both cores were sampled from a flat basin within 4 to 6 m of each other. Isotope analysis showed an exponential decay curve for ^210^Pb (Figure 4) confirming that the sediments were relatively unmixed. However, due to naturally occurring low levels of ^210^Pb in this region, typical ^210^Pb‐based age‐depth models cannot reliably date beyond 3 half‐lives (greater than 70 years, carrying large uncertainties due to instrumental and methodological detection limits). A clear peak in ^137^Cs concentrations at ~13 cm core depth provided a strong indicator of the year 1963 (Figure 4b). Elemental analysis shows a sharp increase in aluminum and titanium (lithogenic particles indicating erosion) beginning at 21 cm, consistent with the timing of the diversion in 1939 at this time (between 19 and 21 cm) (Figure 4c,d). Although our dating estimates do not provide precise dates for each interval sampled, they present strong evidence that allows pre‐ and post‐diversion timeframes to be established in the sediment sufficient to determine lake sturgeon presence or absence before and after the diversion.

### 3.4 eDNA ddPCR Assay

**FIGURE 5:**
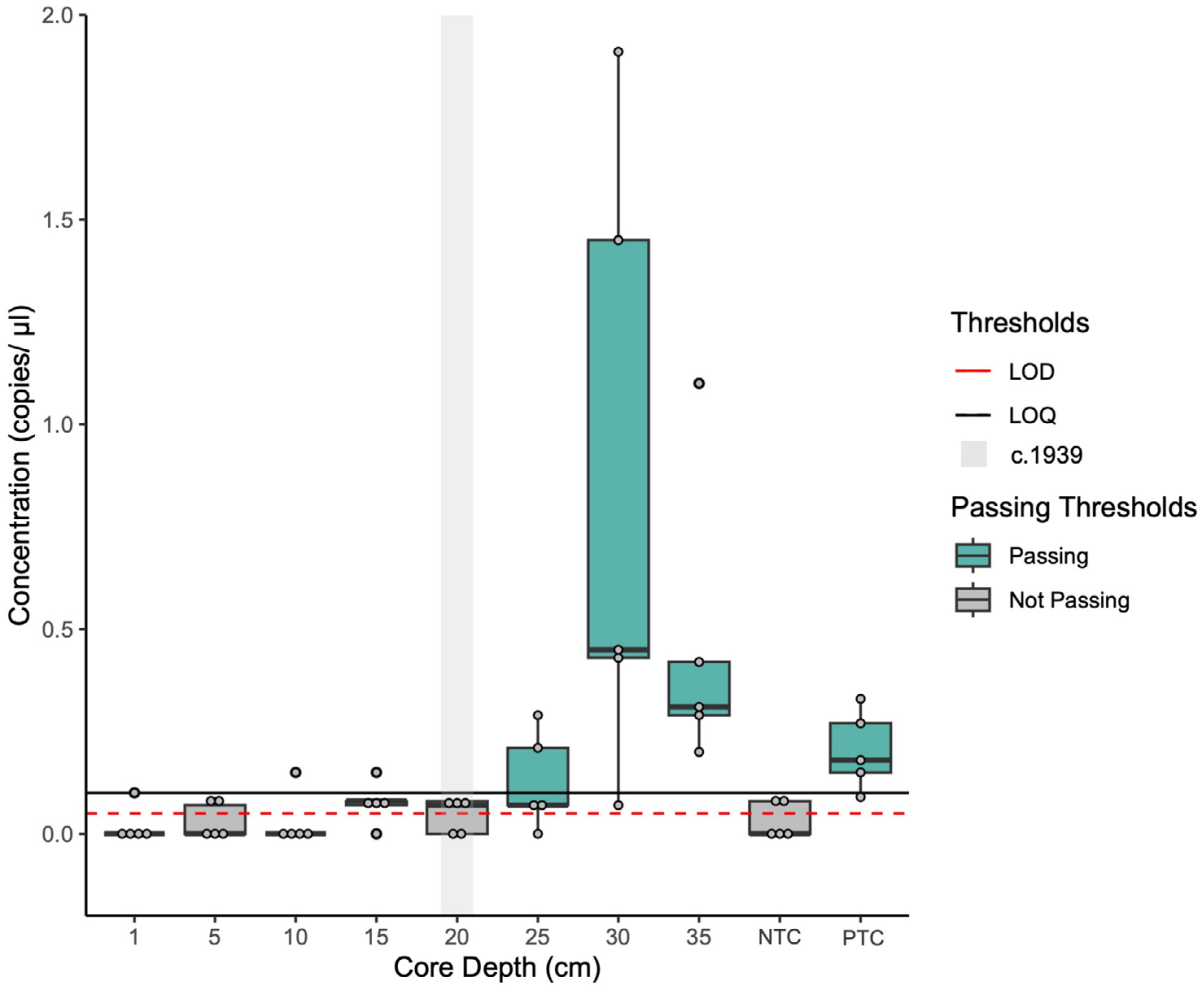
Box and whisker plot of the concentration of lake sturgeon eDNA (copies of DNA/μL) detected in each core section (centimeters (cm) below surface) using digital droplet PCR (ddPCR). Box shows the interquartile range with median line, 95% confidence interval depicted by whiskers, and gray dots show the concentration of each individual replicate per sample. Red dashed line depicts the approximate concentration of the limit of detection (LOD) (1 positive droplet in at least 3 of 5 replicates equating to approximately 0.035 cp/uL). Black solid line depicts the limit of quantification (LOQ) of 0.1 cp/μL (see Figure 2). Gray boxes depict core sections with no positive detection (not passing the LOD and LOQ) and blue boxes depict sections with positive detection. Shaded vertical gray area depicts approximately 1938 and the construction of the diversion project (see Figure 3 for information on the core chronology).

Fluorescence thresholds were set independently for each well as the background fluorescence differed by well with an average threshold of 2715.13 ± 146 (Table 1). All NTCs were included; two of the five NTCs had one droplet above the fluorescence threshold and were considered false positives, and the remainder had no positive droplets. All five PTC samples were included with 2.4 ± 1 positive droplets and a mean concentration of 0.20 cp/μL in 14,833 ± 1144 accepted droplets from a 0.4 pg/μL genomic DNA sample from tissue (Table 1). LOQ was set as 0.1 cp/μL, and the LOD was set as 1 positive droplet in 3 of 5 replicates or approximately 0.035 cp/μL (Figure 3). All eDNA samples were included with an average of 16,228 ± 2114 accepted droplets (Table 1). eDNA samples from core sections 1, 5, and 10 cm [2020–1966] had no detectable DNA with 1, 2, and 2 positive droplets across 5 replicates, respectively (< LOD); samples from core sections 15 and 20 cm [1947–1934] had 5 and 3 positive droplets, respectively, and were considered detectable but not quantifiable (> LOD, < LOQ); sections 25, 30, and 35 cm [~1925–1910] were quantifiable (> LOQ) with 9, 57, and 33 positive droplets and a concentration of 0.13, 0.85, and 0.47 cp/μL respectively (Table 1; Figure 5).

**TABLE 1:** Digital Droplet PCR QX manager 2.0 output and calculated limit of detection (LOD) and quantification (LOQ). Each row is the merged data from all replicates for each core section and negative template control (NTC) and positive template control (PTC). Columns are listed as CFCS modeled date from the isotope data in Figure 3, average DNA concentration (cp/μL), passing limit of detection, passing limit of quantification sum of positive droplets, sum of negative droplets, average fluorescence threshold, mean amplitude of positive droplets, and the mean amplitude of negative droplets. A 20 cm depth is the closest core section to the 1939 diversion event. Data for individual replicates can be found at https://figshare.com/s/aa2bc0ba1d1c43fddc6e.

Core septh (cm)	CFCS date	Concentration (cp/μL)	LOD	LOQ	Positive droplets	Accepted droplets	Negative droplets	Fluorescence threshold	Mean amplitude positives	Mean amplitude negative
1	2020.13	0.02	No	No	1	82,450	82,449	3000.00	20,884.95	2224.22
5	2001.63	0.03	No	No	2	84,465	84,463	2705.91	3935.89	2256.83
10	1965.77	0.03	No	No	2	81,738	81,736	2727.59	3016.48	2282.87
15	1947.17	0.08	Yes	No	5	82,401	82,396	2761.82	2984.89	2309.46
20	1934.48	0.04	Yes	No	3	83,902	83,899	2680.79	2732.41	2283.79
25	1925.29	0.13	Yes	Yes	9	84,917	84,908	2628.33	2827.36	2287.61
30	1917.53	0.85	Yes	Yes	57	84,372	84,315	2589.16	2645.52	2297.50
35	1909.69	0.47	Yes	Yes	33	88,590	88,557	2608.13	2695.86	2328.09
Negative	N/A	0.06	No	No	2	38,920	38,918	2955.67	2276.08	2352.84
Positive	N/A	127.65	Yes	Yes	4744	49,159	44,415	2600.99	4694.14	2267.47

## 4 Discussion

Knowledge from Ginoogaming and #58 First Nations highlighted the negative trend in the *Namaay* population over the last ~100 years as industry‐driven human impacts on the system have increased. Highlighting the effect of the Long Lake Diversion and logging (or more broadly, resource extraction) on the natural balance in the system through increased turbidity, floating debris, and shoreline destruction surrounding the diversion project. The construction of the Kenogami Control Dam brought concern for the migration of *Namaay* into the UKW and specifically *Ginoo‐gami*, altering integral parts of their life cycle (feeding and spawning) (Figure 2). Observations after the construction saw the *Namaay* population disappear from the UKW and *Ginoo‐gami* as migrating individuals were impeded by the dam. Our eDNA assays mirror this, showing clear eDNA detections up to just after dam construction, but none thereafter (Figure 5). Ginoogaming and #58 First Nations Knowledge holders indicated that the population continues to spawn on gravel beds just below the dam and adjacent lakes and rivers, still connected to Hudson Bay in small numbers. The presence of spawning individuals suggests potential for reintroduction into *Ginoo‐gami* if the dam were removed or passage through were actively facilitated. Removal of the dam (or means for passage through) and revitalization of *Namaay* into the UKW and *Ginoo‐gami* would also contribute to the revitalization of *mino‐biimadiziwin* (the physical, spiritual, and cultural health of the community) by supporting their active relationship with this *nadzookanak* fish and each other.

ITK here provides us with a documented ecological history of the lake sturgeon population in the UKW along with factors that have coincided with their population decline, providing avenues for further research. By developing a study guided by knowledge from Ginoogaming and Long Lake #58, we were able to evaluate temporal trends in lake sturgeon presence in Long Lake over the last 120 years using sedDNA. The decline in lake sturgeon sedDNA strongly aligns with the increased industrial activity in the early 1900s and the construction of the Long Lake Diversion Project. The relative abundance of lake sturgeon sedDNA began to decrease above the 30 cm interval sample (in the sediment core) down to an unquantifiable value around 20 cm, the time when the 1939 Diversion Project likely occurred (Figure 5), based on elemental analysis (Figure 4). The data suggest that some lake sturgeon may have persisted in Long Lake after the dam was constructed, as we detected sedDNA in the 15 cm interval of the core dated to the late 1940s–50s (Figures 4 and 5). Although this was below our limit of quantification, these data may represent true detections as some individuals may have been trapped above the dam in low numbers, an assertion supported by ITK of remnant *Namaay* presence in Long Lake after the diversion. However, this presence may also indicate stochasticity in eDNA sedimentation and adsorption onto sediment particles or from smearing during sample collection (Pietramellara et al. 2009). Other studies have found quantitative PCR and eDNA to be effective in detecting fish species in sediment cores (Turner et al. 2015; Lopez et al. 2023), although these have often focused on abundant and non‐migratory species which likely increased the sedDNA signal (Nelson‐Chorney et al. 2019; Kuwae et al. 2020; Lopez et al. 2023). The use of ddPCR generally increases the accuracy and precision in detecting and quantifying fish eDNA at very low abundance in water samples (Doi et al. 2015; Baker et al. 2018; Brys et al. 2021) and has recently been used to detect eDNA in sediment (Hamaguchi et al. 2018; Thomson‐Laing et al. 2023). ddPCR has further proven to reduce the effect of inhibition in environmental samples and the occurrence of false positive and negative detections (Doi et al. 2015; Verhaegen et al. 2016; Guri et al. 2024). This has facilitated studies using only one replicate to determine species presence and as little as one positive droplet in 20,000 (Thomson‐Laing et al. 2023; Guri et al. 2024). We have included multiple replicates as low‐abundance DNA may not be uniformly distributed in the environment and our extracted sample (Barnes et al. 2021; Tournayre et al. 2024). Our results do align with the detection limits of other studies using eDNA and ddPCR (Brys et al. 2021; Tournayre et al. 2024).

We demonstrate the value of oral traditions in identifying, understanding, and responding to the impacts of historical industrialization on a natural ecosystem and the potential for co‐produced knowledge alongside sedimentary eDNA to evaluate species with histories undocumented by Western science. Indigenous people have monitored and cared for their natural environments for millennia; however, recent large‐scale industrialization, natural resource projects, and colonization have made conservation with traditional methods alone challenging. Similarly, Western management continues to fall short in comprehending and mitigating the impacts of human encroachments on ecosystems. Large‐scale diversion projects are widely known to negatively impact aquatic life (Piazza and La Peyre 2011; Xiang et al. 2022; Pringle et al. 2000). Projects like the Long Lake Diversion Project that were constructed before environmental assessment standards and without proper consultation with local Indigenous communities must be evaluated for direct and historical impacts on aquatic ecosystems, and mitigation plans should be put into place to repair the damages they have (and continue to have) caused. By drawing on the strengths of both ITK and molecular ecology, we uncovered the historical population decline of lake sturgeon in Long Lake and the UKW coincident with dam construction.

## 5 Conclusion

Our study shows that the local decline in the lake sturgeon is associated with the Long Lake Diversion project, as we saw lake sturgeon sedDNA decline then disappear in sediment deposited after dam implementation, similar to Indigenous communities' record of occasional sightings after the dam, but then an eventual full absence. This is the first stage of what should be a broad research effort seeking to understand the totality of impacts that the diversion had on this system. Demographic insights from the remaining lake sturgeon population below the dam will help us to understand the current population status and potential for reintroduction into Long Lake through actions such as the inclusion of a fish ladder and community‐run hatchery. Analysis of additional biotic and abiotic changes that occurred will enrich our understanding of the impacts of the Long Lake Diversion Project as well as potential impacts of other systems that have been subject to large‐scale habitat alterations and resource extraction. More broadly, continuing to acknowledge and consider ITK alongside scientific research will help us identify unrealized consequences of development on ecosystems and their constituent species, as well as the impacts on communities that live in reciprocity and harmony with nature according to natural law, as they have since time immemorial. ITK strengthens such studies with insights and data accrued over long temporal scales, but also provides holistic and impactful conservation actions for future sustainability.

## Author Contributions

**Stafford Rohtehrá:kwas Maracle:** conceptualization (equal), data curation (equal), formal analysis (lead), methodology (lead), validation (equal), writing – original draft (lead), writing – review and editing (equal). **Monica L. Garvie:** conceptualization (lead), data curation (lead), formal analysis (equal), methodology (equal), validation (equal), writing – original draft (equal), writing – review and editing (equal). **Calvin Taylor Sr.:** data curation (equal), validation (equal), writing – review and editing (equal). **Michael Fisher:** data curation (equal), validation (equal), writing – review and editing (equal). **Brian F. Cumming:** funding acquisition (equal), project administration (equal), resources (equal), supervision (equal), writing – review and editing (equal). **Stephen C. Lougheed:** funding acquisition (equal), project administration (equal), resources (equal), supervision (equal), writing – review and editing (equal).

## Conflicts of Interest

The authors declare no conflicts of interest.

## Data Availability

All associated data for Figures 2, 3, 4, 5, and Table 1 are available on figshare (link: https://figshare.com/s/aa2bc0ba1d1c43fddc6e).

## Endnotes

^1^
Anishinaabemowin (Ojibwe language) words in this paper follow local spelling conventions as conveyed to authors directly by knowledgeable community first speakers.

^2^
Several regional spellings exist; “Anishinabe” is the spelling M. Fisher (and many others) prefers when referring to his identity. The spelling “Anishinaabe” is used when referring to M. Garvie's identity and Anishinaabe peoples in general following the phonetics convention of the Ojibwe People's Dictionary (https://ojibwe.lib.umn.edu/).
